# Atherosclerosis imaging with ^18^F-sodium fluoride PET: state-of-the-art review

**DOI:** 10.1007/s00259-019-04603-1

**Published:** 2019-11-27

**Authors:** Poul F. Høilund-Carlsen, Michael Sturek, Abass Alavi, Oke Gerke

**Affiliations:** 1 Department of Nuclear Medicine, Odense University Hospital, Odense, Denmark; 2 Research Unit of Clinical Physiology and Nuclear Medicine, Department of Clinical Research, University of Southern Denmark, Odense, Denmark; 3 Department of Anatomy, Cell Biology, Physiology, Indiana University School of Medicine, Indianapolis, IN USA; 4 Department of Radiology, Perelman School of Medicine, University of Pennsylvania, Philadelphia, PA USA

**Keywords:** Atherosclerosis, PET, ^18^F-sodium fluoride, NaF, Calcification, Quantification

## Abstract

**Purpose:**

We examined the literature to elucidate the role of 18F-sodium fluoride (NaF)-PET in atherosclerosis.

**Methods:**

Following a systematic search of PubMed/MEDLINE, Embase, and Cochrane Library included articles underwent subjective quality assessment with categories low, medium, and high. Of 2811 records, 1780 remained after removal of duplicates. Screening by title and abstract left 41 potentially eligible full-text articles, of which 8 (about the aortic valve (*n* = 1), PET/MRI feasibility (*n* = 1), aortic aneurysms (*n* = 1), or quantification methodology (*n* = 5)) were dismissed, leaving 33 published 2010–2012 (*n* = 6), 2013–2015 (*n* = 11), and 2016–2018 (*n* = 16) for analysis.

**Results:**

They focused on coronary (*n* = 8), carotid (*n* = 7), and femoral arteries (*n* = 1), thoracic aorta (*n* = 1), and infrarenal aorta (*n* = 1). The remaining 15 studies examined more than one arterial segment. The literature was heterogeneous: few studies were designed to investigate atherosclerosis, 13 were retrospective, 9 applied both FDG and NaF as tracers, 24 NaF only. Subjective quality was low in one, medium in 13, and high in 19 studies. The literature indicates that NaF is a very specific tracer that mimics active arterial wall microcalcification, which is positively associated with cardiovascular risk. Arterial NaF uptake often presents before CT-calcification, tends to decrease with increasing density of CT-calcification, and appears, rather than FDG-avid foci, to progress to CT-calcification. It is mainly surface localized, increases with age with a wide scatter but without an obvious sex difference. NaF-avid microcalcification can occur in fatty streaks, but the degree of progression to CT-calcification is unknown. It remains unknown whether medical therapy influences microcalcification. The literature held no therapeutic or randomized controlled trials.

**Conclusion:**

The literature was heterogeneous and with few clear cut messages. NaF-PET is a new approach to detect and quantify microcalcification in early-stage atherosclerosis. NaF uptake correlates with cardiovascular risk factors and appears to be a good measure of the body’s atherosclerotic burden, potentially suited also for assessment of anti-atherosclerotic therapy.

**Electronic supplementary material:**

The online version of this article (10.1007/s00259-019-04603-1) contains supplementary material, which is available to authorized users.

## Introduction

Atherosclerosis is the world’s number one killer [1]. Most of what we do against it are late occurring examinations and interventions that cannot effectively counteract the disease. What is needed is the opposite, i.e., early diagnosis and grading of the disease when it may still be susceptible to therapy. Molecular in vivo imaging offers a new approach for studying early-stage atherosclerosis by means of positron emission tomography (PET) with two of the most widely used tracers, ^18^F-fluorodeoxyglucose (FDG) and ^18^F-sodium fluoride (NaF), markers of inflammation and ongoing ossification, respectively. This is relevant because the two processes are closely associated with the beginning of atherosclerosis and, thus, may provide a window for the detection and measurement of early-stage atherosclerosis. FDG-PET imaging of atherosclerosis was introduced in 2001 [2, 3] and NaF-PET imaging approximately 10 years later [4, 5]. While FDG-PET in atherosclerosis has been reported in multiple publications, papers on NaF-PET imaging in atherosclerosis are less numerous. It has gradually become clear that FDG uptake is not a straightforward marker of atherosclerosis, whereas a number of observations point to NaF uptake as being of potential clinical importance in atherosclerosis and a means of obtaining a better understanding of its pathophysiologic mechanism. The purpose of this review is to establish an overview of published original research of clinical relevance about NaF-PET in atherosclerosis and to examine what new information has been provided thus far and which important issues should be in focus of future research.

## Materials and methods

### Literature retrieval

The principles of a systematic review were used in an extensive literature search of PubMed/MEDLINE, Embase, and Cochrane Library to extract relevant peer-reviewed articles on NaF-PET imaging in atherosclerosis. The search algorithms were based on two combinations of search terms of which every included article fell into at least one of these combinations:

- Sodium fluoride AND atherosclerosis;
- Sodium fluoride AND (inflammation OR calcification) AND (heart, arteries, OR aorta).

The search included articles on humans published in English until December 31, 2018. The PICOS approach (population, intervention, comparison, outcome, study design) was adopted [6] and comprised the following:

- Patients with any disease provided that NaF-PET was used for the detection of atherosclerosis
- Studies with focus on diagnostic performance, lesion detection, qualitative evaluation, and feasibility
- Studies in which the stated primary aim was an evaluation of atherosclerosis (no restriction on comparator methods if any)
- No restriction on outcome measures
- No restriction on study design.

**Fig. 1:**
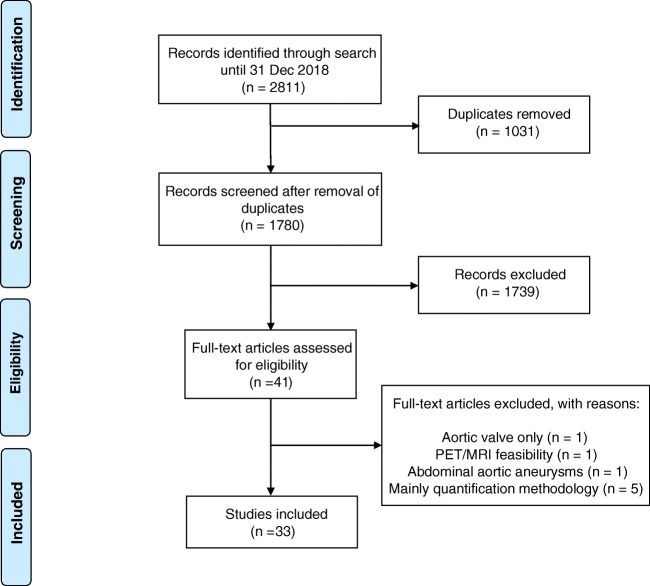
PRISMA flow diagram

Records from the databases were transferred to the Endnote reference tool to identify and remove duplicates and book sections and screened by title and abstract according to strict inclusion and exclusion criteria by one senior researcher (OG). Exclusion criteria comprised the following: (a) articles outside the scope of this review; (b) editorials, letters, comments, or conference proceedings; and (c) case studies, studies with other tracers than NaF, pure methodology studies, and review articles. After removal of duplicates, one researcher, experienced in clinical physiology and nuclear medicine and in cardiology (PFHC), reviewed titles and abstracts and checked their eligibility. This researcher also reviewed the full-text versions of preliminarily included articles and decided in- or exclusion. The selection procedure is illustrated in a Preferred Reporting Items for Systematic Reviews and Meta-Analyses (PRISMA) diagram [7] (Fig. 1), which mirrors the result of the conducted systematic literature search. Data were extracted from each of the selected articles and grouped according to number, age, and type of patients, prospective or retrospective data collection, tracer (NaF only or NaF and FDG), and arteries studied.

#### Quality assessment

Articles were categorized by a simple subjective a priori system with three categories: low, medium, or high quality. The system was similar to the QUADAS-2 criteria, which is based on answers “yes,” “no,” or “unclear” to 14 specific questions [8]. We evaluated based on each article’s ability to sufficiently meet 12 quality items, which were given a grade, where 0 = no fulfillment (complete lack of mention), 1 = partly fulfillment (insufficient description or documentation), or 2 = complete or nearly complete fulfillment (sufficient and relevant documentation or argumentation). The items were as follows: (1) clinical relevance, (2) aim(s), (3) hypothesis, (4) prospective or retrospective data collection, (5) design and power, (6) description and size of material(s), (7) description and use of method(s), (8) statistical analysis, (9) relevant presentation of results, (10) interpretation, (11) limitations, (12) conclusion(s). The maximum obtainable score was 12 × 2 = 24. Quality was designated low (score 0–8), medium (score 9–16), or high (score 17–24).

## Results

Out of 2811 initial records, 41 full-text articles remained for assessment of eligibility. Eight of these were dismissed for reasons stated in Fig. 1. Thus, 33 human studies were included in the qualitative synthesis of evidence, of which 6, 11, and 16 were published in 2010–2012, 2013–2015, and 2016–2018, respectively. Eight papers dealt exclusively with the coronary arteries [9–16] and seven solely with the carotid arteries [5, 17–22], while only one article focused on the thoracic aorta [23], the infrarenal aorta [24], and the femoral arteries [25]. Four studies were looking on the whole heart and aorta [26–29], whereas the remaining 11 articles focused also on more than one part of the cardio-arterial system, i.e., coronaries, carotids, and aorta [30–32]; coronaries, aorta, and femoral arteries [33]; coronaries and carotids [34]; carotid, aorta, iliac, and femoral arteries [4, 35]; carotid, aorta, femoral arteries [36]; carotid, aorta, iliac [37]; carotid, subclavian, iliac, whole aorta [38]; and, finally, aorta and iliac arteries [39].

In 24 articles, NaF was the only PET tracer, whereas 9 compared NaF with FDG in the same patients [9, 10, 19, 21, 23, 30, 34, 36, 37]. Thirteen articles were retrospective analyses of PET scans performed for other purposes, typically search for metastases in cancer patients, whereas 20 papers described prospectively collected materials, but seldom in a design suited to answer a specific hypothesis about atherosclerosis. Eight articles reported data from ≤ 25 patients (range 4–25), while 25 papers included results from 26–409 subjects or patients. According to our subjective quality system, one study was of low quality (score = 7), 14 of medium (score range 11–16), and 18 of high quality (score range 17–23). Below (and in Tables 1, 2, 3, and 4, Supplementary material) the literature content is summarized in 6 sections, each comprising results from some of the 33 included articles illustrating:

- disease mechanisms and targeting [14, 15, 17, 22, 24],
- early detection and prevalence of NaF uptake in the heart and major arteries [4, 13, 18, 19, 24, 26, 31, 33, 35, 36],
- NaF uptake in vulnerable, high-risk, and ruptured plaques [10, 12, 14–16, 20, 21, 34],
- influence of age, sex, and other factors on NaF uptake [5, 25–28, 30, 31],
- association between NaF uptake and cardiovascular risk factors [9, 11, 23, 29, 32, 35, 38],
- NaF uptake and progression of atherosclerosis [37, 39].

Thus far, no intervention studies and no randomized clinical trials aiming to reverse NaF-avid microcalcification have been published.

### Disease mechanisms and targeting

**Fig. 2:**
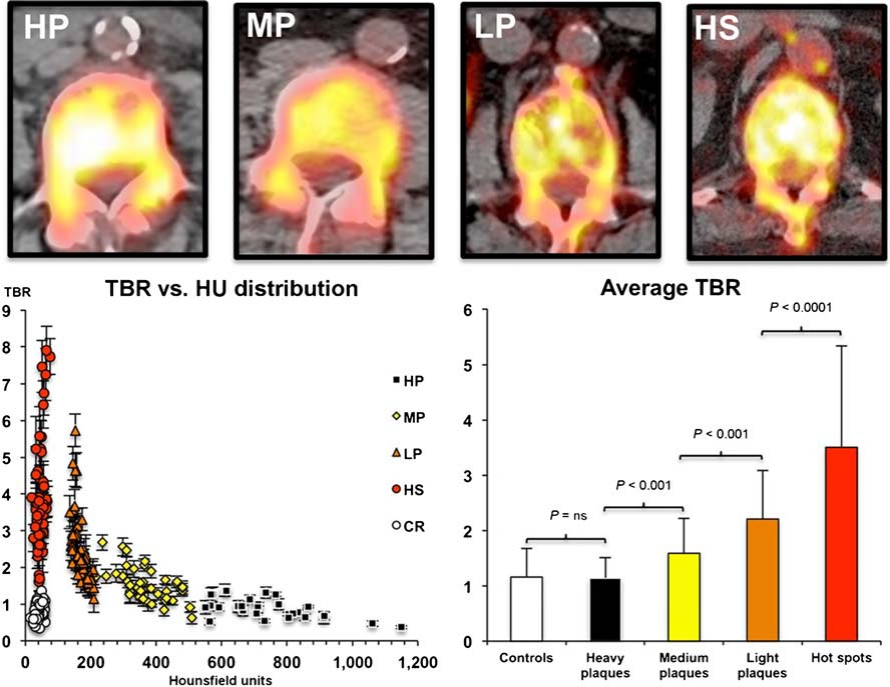
Inverse relation between arterial wall uptake of NaF and CT-visible calcification in the infrarenal aorta. High NaF uptake in aortic lesion without CT-calcification (Hot spots = HS) and decreasing NaF uptake with increasing density of CT-calcifications until same low NaF uptake in high density plaques (Heavy plaques = HP) than in controls (reproduced by permission from reference 24)

Irkle et al. demonstrated in carotid endarterectomy samples that fluoride adsorbs to calcified areas in mineralized vascular tissues and that NaF radioactivity is confined to calcification and not to soft tissues. Furthermore, the NaF signal depends on the surface area of calcification as NaF adsorbs only to the outer layer of macrocalcifications in contrast to microcalcifications which have a greater surface area and no barriers to penetration of the tissues resulting in high levels of NaF adsorption [17]. They confirmed this by autoradiography, ex vivo μPET/μCT, and in vivo clinical PET/CT in four patients scanned with NaF-PET/CT before carotid endarterectomy and concluded that “areas of ^18^F-NaF uptake are reporting underlying microcalcifications, which are undetectable by CT” and further that NaF-PET/CT is “the only currently available platform that can non-invasively detect microcalcification in active unstable atherosclerosis” [17]. Fiz et al. evaluated NaF uptake in the infrarenal aorta of 64 patients with breast or prostate cancer and at least one CT-visible (> 130 Hounsfield Units (HU)) calcification and found average target-to-background ratios (TBRs) that were clearly highest (3.6) in arterial “hot spots,” i.e., areas of NaF uptake with no calcification (present in 86% of patients) and decreased stepwise in plaques with light, medium, and heavy density, defined by HU (Fig. 2) [24]. Similarly, Li L et al. found in 32 patients with symptomatic coronary artery disease a decline in maximal TBR from coronary fibrocalcific via thincap with spotty calcifications to thickcap mixed atheroma (from 1.42 via 1.32 to 1.28, respectively) and to normal uptake (0.96) in fibrotic plaque indicating that NaF uptake most likely localizes in the border zone of intensive calcification [15]. Zhang et al. observed in eight patients undergoing carotid endarterectomy significant positive correlation between NaF uptake and calcification in histological sections of carotid plaques, a negative correlation with smooth muscle staining, and no correlation with carotid artery stenosis, HU value, or inflammation [22]. Partly in line with this, Kitagawa et al. reported that per patient in 32 patients with known or suspected coronary artery disease, TBRmax correlated positively with a logarithmically transformed coronary calcification score, while per lesion, partially calcified plaque showed higher TBRmax than calcified and non-calcified plaque, i.e., 1.17 vs 1.00 vs 0.92 [14].

### Early detection and prevalence of NaF uptake

Derlin et al. demonstrated (Table 1, Supplementary material) in their 2010 study of 75 patients, referred to exclusion of bone metastases, CT-calcification at 1930 sites in 84% of patients, NaF uptake in only 254 sites in 76% of patients, co-localization of NaF accumulation, and CT-calcification in 223 areas of uptake (88%), and that only 12% of all arterial calcification sites showed NaF uptake at least in some part [4]. In a large retrospective study of oncologic patients, the same group demonstrated that around 77% of lesions with marked NaF uptake and only about 15% of lesions with FDG accumulation were co-localized with arterial CT-calcification and that coincident uptakes of both NaF and FDG were present in only 6.5% of 215 arterial lesions with radiotracer accumulation in the carotid arteries, the aorta, and femoral arteries [36]. In the third study of 409 oncologic patients, they focused in particular on linear NaF uptake in the femoral arteries and found this in nearly 40% of patients, of which less than half had medial-type linear CT-calcifications [35]. Moreover, they reported CT-visible arterial plaques in carotids, aorta, right and left iliac, left and right femoral arteries present at 3767 sites in 83.1% of the patients aged 19.7–90.8 years, highest in the abdominal aorta, followed by iliac arteries and thoracic aorta. NaF uptake in femoral arteries correlated with multiple risk factors and increased with the number of risk factors, from 9.7% of cases in their group with 0 risk factors to 64.1% in their group with most, i.e., ≥ 5, risk factors [35].

Beheshti et al. demonstrated in a retrospective analysis of 51 oncologic patients a positive linear relation between NaF uptake in the heart and aorta measured in five age groups (≤ 40, 41–50, 51–60, 61–70 > 70 years) and noted that the ≤ 40 age group had a NaF uptake in the heart that was 57% of that of the > 70 age group and in the aorta that was 65% of that of the > 70 age group [26]. Li Y et al. reported in 61 patients examined for bone lesions that NaF uptake was more frequent in the aorta and femoral arteries than CT-calcification and vice versa in the carotid and coronary arteries [33]. Fiz et al. demonstrated in their study that early-stage CT-visible calcification in the abdominal aorta has higher NaF uptake than the late stage calcification [24]. Quirce et al. noted in two small studies higher NaF uptake in patients with symptomatic than asymptomatic carotid plaques [18, 19], while Kitagawa et al. in patients with coronary artery disease found slightly higher NaF uptake in partially than non-calcified and calcified coronary plaques [13]. Finally, Ferreira et al. observed similar NaF uptake in the two sexes, despite the same age level, in the coronaries, carotids, and the aorta of patients with a 10-year risk of fatal events ≥ 5% [31].

### NaF uptake in vulnerable, high-risk, and ruptured plaques

This has been studied in the coronary and carotids arteries only (Table 2, Supplementary material). In a study of 40 patients with acute myocardial infarction, 40 patients with stable angina who underwent invasive coronary angiography and 12 patients (of which 9 were evaluable) undergo who underwent carotid endarterectomy for symptomatic carotid artery disease. Joshi et al. reported slightly, but significantly, higher median NaF TBRmax (1.66 vs 1.24) in culprit lesions compared with non-culprit carotid lesions [34]. Marked NaF uptake was noted at all carotid plaque ruptures and plaques with histologic evidence of active calcification, macrophage infiltration, apoptosis, and necrosis. Moreover, 18% of stable angina pectoris patients had coronary plaques with focal NaF uptake that was associated with more high-risk features on intravascular ultrasound than those without uptake, which made the authors conclude that NaF-PET/CT “is the first non-invasive imaging method to identify and localize ruptured and high-risk coronary plaque” [34]. Lee at al. reported from coronary angiography in 51 patients that NaF uptake, measured as the maximal standardized uptake value (SUVmax) in proximal coronary arteries, was higher in patients with plaques considered high-risk than low-risk by intravascular ultrasound (IVUS) or optical coherence tomography [12]. Li et al. found in 32 patients, most of which with unstable angina, that coronary NaF uptake was associated with high-risk plaque features on IVUS, and that NaF uptake was highest in lesions with the least calcification [15]. In 18 patients with culprit carotid stenosis awaiting endarterectomy and 8 controls without culprit carotid atheroma, Vesey et al. found, using logarithmic SUVmean values, slightly, but significantly, increased NaF uptake in “clinically adjudicated culprit plaques” compared with asymptomatic contralateral plaques which again was clearly higher than in controls (0.29 vs 0.23 vs 0.12). FDG uptake was increased in 7 of 16 culprit lesions but with no difference between culprit vs contralateral plaques or control patients [21]. Kitagawa et al. reported small, but significant, differences in coronary NaF uptake among patients with and without later coronary events, which at 2-year follow-up was 1 myocardial infarction, 3 cases of unstable angina, and 7 coronary revascularizations in 32 patients, and concluded NaF-PET/CT has “the potential to detect high-risk coronary disease and individual coronary lesions and predict future coronary events when combined with cardiac CT” [14]. Marchesseau et al. observed in 10 patients examined 9–24 days after ST-elevation myocardial infarct that TBRmax was significantly higher (2.11 vs 1.36) in culprit than non-culprit lesions and noted also that NaF uptake was clearly higher in scarred than in remote myocardial tissue (0.87 vs 0.72) [16]. Finally, in an experimental study of samples of 17 culprit and 6 non-culprit carotid lesions from 23 stroke patients compared with 15 renal artery samples from healthy kidney donors, Hop et al. found by micro-PET that average NaF uptake (SUVmean) was equally high in culprit and non-culprit lesions, but 5 times as high as in normal renal arteries, and that only 10% of carotid CT-calcifications showed increased NaF uptake, while only about 1/3 of NaF foci showed calcification on CT [20].

### Influence of age, sex, and other factors

A handful of studies have statistically demonstrated that arterial wall microcalcification increases slightly, but significantly, with age [5, 25, 26, 30]. Most studies indicated a significant correlation with age [5, 25, 30], and a single study recorded in adults an increase by 75% from age ≤ 40 to age ≥ 70, albeit with a wide scatter indicating that some young adults have moderately high arterial wall NaF uptake, while a number of old people have fairly low uptake [26]. There was no major age difference between sexes with respect to arterial NaF uptake as observed in more than just a single study [5, 26, 30, 31]. Derlin et al. reported a light association between carotid NaF uptake and male sex in their oncologic patients [5], whereas Blomberg et al. noted in healthy adults with an unfavorable cardiovascular profile that female sex was an independent factor of increased coronary artery NaF uptake [11]. In the only prospective study to date reporting FDG and NaF uptake in healthy individuals, Blomberg et al. reported increase in uptake with age in 89 healthy control subjects that was less pronounced for FDG than NaF in that FDG uptake increased with age only in the descending aorta, whereas correlation of increase in NaF uptake and age was found in the ascending aorta, aortic arch, descending aorta, and the coronary arteries (Table 2, Supplementary material) [30]. Among other factors, the same group examined in a single study of 18 angina pectoris patients and 20 healthy subjects scanned 45, 90, and 180 min after injection of NaF that the contrast between arterial wall NaF uptake and blood pool activity was similar with 45 and 90 min acquisition and not improved after 180 min [27]. In another report, they demonstrated that blood activity, injected dose, and the PET/CT system influence arterial NaF uptake [28].

**Fig. 3:**
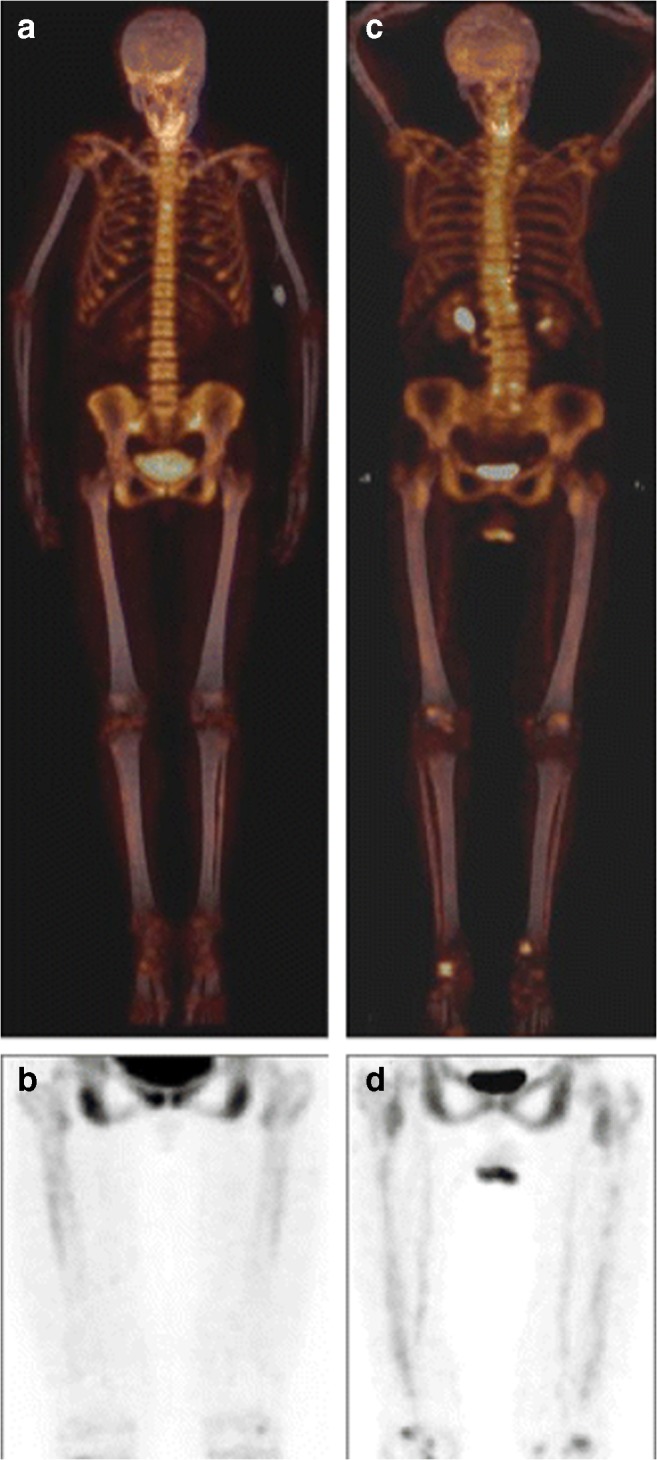
Arterial mineral deposition increases with age, while regional bone metabolism decreases. Coronal fused 18F-sodium fluoride PET/CT (a) and non-fused PET of the thighs (b) of a 29-year-old woman with low arterial mineral deposition (TBR 1.44) and high bone metabolism (SUVmean 8.6). Coronal fused 18F-sodium fluoride PET/CT (c) and non-fused PET of the thighs (d) of a 83-year-old woman showing high arterial mineral deposition (TBR 2.18) and low bone metabolism (SUVmean 6.7) (reproduced by permission from reference 25)

The interesting observation that arterial NaF uptake is in osseous exchange with bones was first reported by Derlin et al., who, in 608 femoral arterial segments of 304 patients (aged 20–91 years) referred to exclusion of bone metastases, found an inverse correlation between arterial NaF uptake and bone metabolism and that arterial mineral deposition increases with age, while regional bone metabolism decreases (Fig. 3) [25].

### Association between NaF uptake and cardiovascular risk factors

**Fig. 4:**
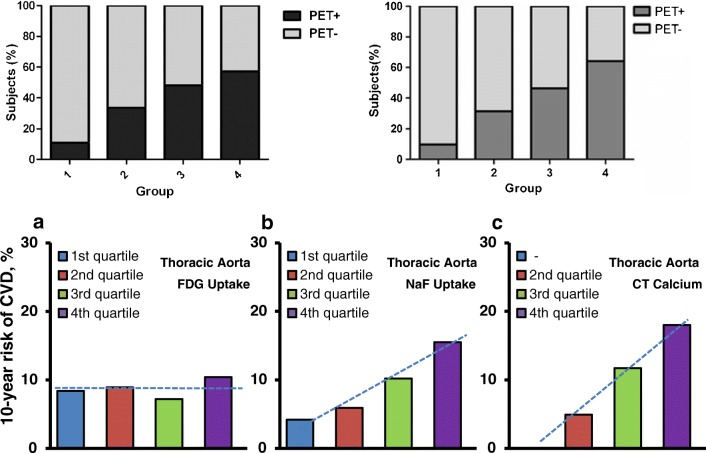
Positive relationship between arterial NaF uptake and cardiovascular risk. Upper panel, left: Relationship between cardiovascular risk factors and presence of NaF uptake in carotid arteries. (reproduced with permission from reference 5). Upper panel, right: Relationship between cardiovascular risk factors and presence of NaF uptake in carotid arteries (reproduced with permission from reference 35). Lower panel: 10-year Framingham risk score in relation to quartiles of (a) thoracic aorta FDG activity, (b) thoracic aorta NaF activity, and (c) thoracic aorta CT calcium burden. The risk is similar in all quartiles of thoracic aorta FDG uptake, but increases linearly with each increasing quartile of thoracic aorta NaF uptake (*P* < 0.001 for a linear trend) and with each increasing quartile of thoracic aorta CT calcium burden (*P* < 0.001 for a linear trend) (modified from reference 23)

From early on, the Derlin group showed in their large retrospective studies significant association between NaF uptake in the carotid arteries and multiple risk factors including age, male sex, hypertension, and hyperlipidemia, but not history of smoking, diabetes, obesity, or cardiovascular events [5] and in the femoral arteries correlation between NaF uptake and the same risk factors plus diabetes and prior cardiovascular events [25, 35] (Tables 1 and 3, Supplementary material). Dweck et al. found that patients with increased coronary NaF activity had higher rates of prior cardiovascular events and angina and higher Framingham Risk Score (FRS) [9]. Morbelli et al. demonstrated in 80 oncologic patients a significant relationship between NaF uptake in the carotid, subclavian, and iliac arteries and the aorta and all risk factors except BMI, while visible CT-calcification was dependent on age only (Table 4, Supplementary material) [38]. Fiz et al. split their 77 patients with breast or pancreatic cancer without major cardiovascular events and without statin therapy into three risk groups according to their 10-year FRS, high, intermediate, and low, and found a clear association between descending aorta and cardiac uptake of NaF and cardiovascular risk and suggested that the analysis approach with a global metabolic score is superior to TBR values from selected foci, since the global score includes NaF uptake in both micro- and macrocalcification [29]. Blomberg et al. reported a similar positive relationship between NaF uptake in the thoracic aorta and FRS as between CT-visible calcification and FRS, but not between FDG uptake and FRS (Fig. 4) [23]. In their prospective CAMONA trial, they demonstrated in 89 healthy controls that coronary NaF uptake increases linearly with the number of cardiovascular risk factors and that female sex, age, and BMI are independent factors of increased coronary NaF uptake (Table 4, Supplementary material) [11]. Finally, Oliveira et al. reported in 25 patients with hypertension, but without clinically apparent cardiovascular disease, that 96%, 40%, and 64% had increased NaF uptake in the aorta, the carotid, and coronary arteries, respectively, and that the 14/25 of their patients, who had ≥ 5 risk factors, had an increased overall uptake of NaF which was positively correlated with predicted fatal cardiovascular risk SCORE and thoracic fat volume, but not with coronary calcium score [32].

### NaF uptake and disease progression

Two of the 33 articles were reports on this topic. In 34 patients (aged 19–78 years, 18 women), Ishiwata et al. examined NaF uptake in the thoracic and abdominal aorta and in the common iliac artery to see if NaF uptake could predict progression of CT-calcification. A total of 182 sites with > 130 HU on CT and 96 hot spots, i.e., NaF-avid spots with < 130 HU at baseline were compared with CT at follow-up after 1 year and later. They noted that baseline NaF uptake did not correlate with CT-calcification in HUs or volume after 1 year, but with change in calcification volume and change in HU score. Novel CT macrocalcification was observed after a mean of 1446 days in 19/96 of original NaF hot spots without macrocalcifiation. Unfortunately, only CT and not NaF imaging was repeated [39]. This was done in a recent study by Li X et al. who analyzed 19 out of 34 multiple myeloma patients (aged 68 ± 9 years, 8 women), who underwent both FDG and NaF imaging at an interval of 15 ± 4 months without in between intervention or important change in medication. TBRmax was reported in three groups of lesions, non-calcified (< 130 HU), mildly calcified (130–399), and severely calcified (≥ 400). NaF and FDG uptake was not correlated in non-calcified lesions, but in mildly calcified (*r* = 0.7) and highly calcified lesions (*r* = 0.4). FDG uptake was higher in non-calcified than calcified lesions, the same pattern for NaF. NaF uptake increased with increased plaque density, whereas FDG uptake decreased. Inflammation and osteogenesis showed concordant changes in 86% of non-calcified, 81% of mildly calcified, and in 47% of highly calcified lesions [37].

## Discussion

### Literature survey

The literature search produced nearly 3000 hits, one-third of which were duplicates, whereas 1739 records were not included because they met one or several exclusion criteria, while finally eight additional papers were excluded for reasons stated in Fig. 1, leaving only 33 included articles. On average, less than 4 new publications per year have been published since the first report by Derlin et al. in 2010 [4], however, with almost a tripling from the first to the last 3-year period. The studies published so far varied in size, purpose, and methodology; several were retrospective analyses of patient populations examined originally for other purposes. Prospective studies with distinct hypotheses, relevant design, proper material of sufficient size, and reproducible methodologies were in short supply. According to our subjective assessment, only a little more than half of the studies were of high quality; several were without hypothesis, had insufficient description of materials and methods, lacked reference standards, and sometimes made conclusions not sustained by the results. Only few papers had a clearly stated, clinically relevant, and patient-related purpose, and randomized controlled trials or intervention studies have not been conducted so far. Due to this diversity, our systematic literature search could not provide an answer to a particular scientific question; we were left to summarize the information about NaF-PET in atherosclerosis that the literature had provided until now and point to important issues that remain unanswered.

### Disease mechanisms and targeting

According to experimental literature, microcalcification, categorized by nodules < 50 μm, heralds the onset of arterial wall mineralization triggered by cell death and inflammation [40–42]. These nodules are too small to be detected by CT imaging, but since they are the building stones of macrocalcification, defined as nodules ≥ 50 μm [43] and can be detected by NaF-PET imaging, they have become of significant interest. In line with this, Irkle et al. highlighted the specificity of NaF uptake, its confinement to microcalcification and not tissue, and pointed to NaF uptake as a marker of nascent calcification and for testing of the efficacy of anti-atherosclerotic interventions [17].

The literature tells us that FDG-avid foci in the arterial wall are very common, with and without CT-calcification (Table 1, Supplementary material), and something that ”waxes and wanes” at short intervals [44] and therefore perhaps a natural inflammatory response to arterial injury. In contrast, arterial wall NaF uptake appears to be a more stable process localized in, but not as part of, tissue, predominantly muscular necrosis. In line with this, Marchesseau and co-workers demonstrated by both PET/CT and PET/MRI imaging in patients with recent acute myocardial infarction significantly higher (+ 45%) NaF uptake in culprit than non-culprit coronary lesions and higher (+ 14%) uptake in infarcted than non-infarcted myocardium [16].

### Early detection and prevalence of NaF uptake

**Fig. 5:**
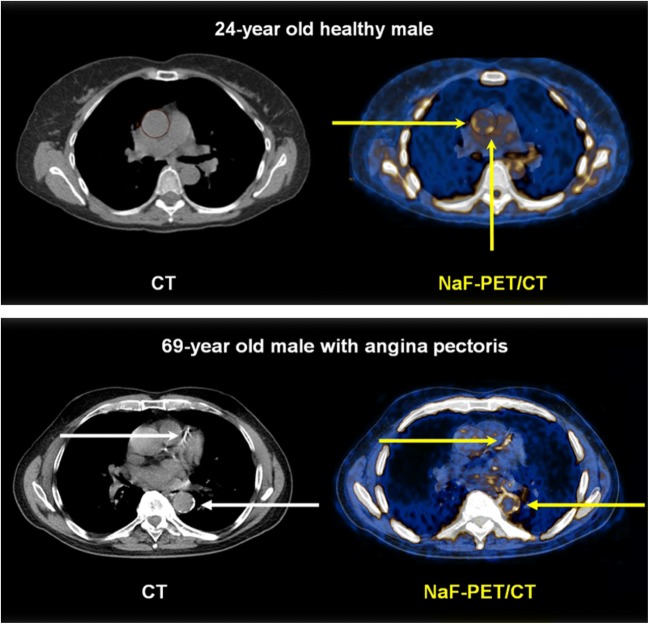
Abnormal arterial wall NaF uptake has been observed down to the age of 20 and is most often present in the absence of CT-calcification. Upper panel: NaF uptake (right) in the thoracic aorta (yellow arrows) of a 24-year-old symptom-free male with no CT-visible calcification (left). Lower panel: 69-year-old male with angina pectoris and CT-visible calcification in the left anterior descending coronary artery and the descending thoracic aorta (white arrows, left) and NaF uptake in the same arteries, incongruent with CT-calcifications and with far greater circumferential extension in the aorta (yellow arrows, right) (images from material of references 23 and 30)

**Fig. 6:**
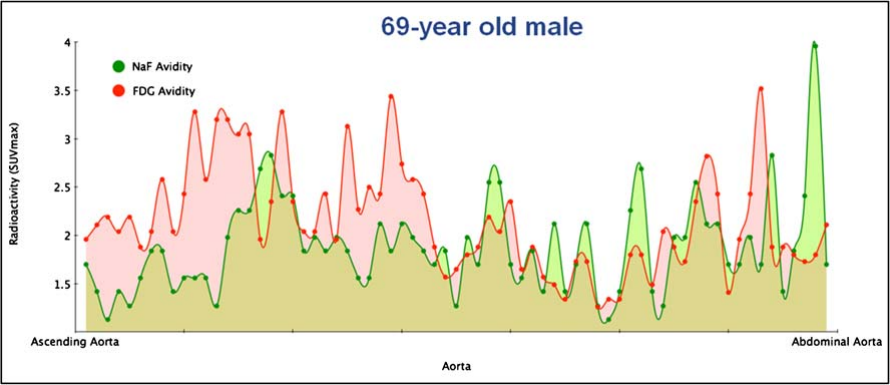
NaF uptake is often not confined to identifiable lesions, but varies through each arterial segment. Variation in FDG (red areas) and NaF (green areas) uptake through the entire aorta. The red and green dots are blood background subtracted SUVmax values from horizontal slices of 3.75 mm thickness. Note the outspoken disconcordance and the generally higher FDG uptake in the thoracic part (to the left) and generally higher NaF uptake in the abdominal part of the aorta (to the right) (from the material of reference 23)

The studies demonstrate localized, discrete, or diffuse abnormal arterial wall uptake in men and women down to the age of 20 (Fig. 5) and that NaF uptake is not confined to identifiable lesions, but varies considerably through each arterial segment (Fig. 6) [23, 30, 36]. Studies in a metabolic syndrome pig model suggest that increased coronary uptake of NaF is present in the very early-stages of atherosclerosis since fatty coronary streaks of these swine pick up NaF [45]. Pathologic studies have demonstrated that almost every North American child over the age of 3 years has some degree of aortic fatty streaks [46] and that by the age of 15–19 years, fatty streaks occupy about 25% of the aortic intima in the thoracic and abdominal aorta and that they increase in the abdominal aorta to occupy nearly 40% at the age of 34–40 years [47]. There is indication that fatty streaks may translate into raised lesions of atherosclerosis (a collective term for fibrous plaques and associated complications) and that coronary events become frequent in a population when the average extent of coronary raised lesions in middle-aged persons approaches 30% of the coronary intimal surface [47]. Considering the apparent lack of difference in arterial NaF uptake in women and men [5, 26, 30, 31], it is suggestive that in age groups 15–19 to 30–34 years, fatty streaks have been found to be equally frequent in the coronary arteries of men and women, but that women have about one-half of the extent of raised lesions at all ages. Furthermore, the thoracic aorta is highly susceptible of fatty streaks, but not to raised lesions, and that abdominal aorta of women has more extensive fatty streaks than that of men, but an equal extent of raised lesions [47].

### NaF uptake in vulnerable, high-risk, and ruptured plaque

Several authors have with different quantification algorithms demonstrated higher NaF uptake in vulnerable, high-risk, and ruptured plaques, which may not be, as often claimed [10], a means to accurately identify high-risk plaques or predict future cardiovascular events in individual patients (Table 2, Supplementary material). To be of clinical use, identification of individual high-risk plaques and prediction of coming events should be possible in single patients with a fairly high probability as for instance one of 85% or higher, which has so far not been demonstrated. Moreover, the search for presumed vulnerable plaques may not be as useful as often stated because, as suggested by Arbab-Zadeh and Fuster, a state of generalized vulnerability may be more important overall than characterizing the individual sites of vulnerability in the individual patient, the reasons being that plaque rupture often occurs without clinical symptoms, plaque morphology changes over a few months, and plaque rupture frequently occurs apart from the culprit lesions [48]. These are insightful perspectives and suggest at least three major concepts. (1) Generalized vulnerability may be the earliest molecular calcification, preceding microcalcification, and should be studied in preclinical models to determine the basis. Molecular calcification would almost certainly occur before subclinical plaque rupture. (2) The natural course of focal NaF uptake must be studied prospectively to determine whether these sites become sufficiently vulnerable to trigger acute myocardial infarction requiring coronary intervention, e.g., stenting. This would add further clarity to the prospective study of Joshi et al. [34]. (3) Continued use of artery-specific 18F-NaF imaging with coronary CT angiography [45, 49, 50] and a more high throughput global scoring system [26] not requiring radiopaque contrast infusion may be highly relevant for coronary artery risk stratification and prospective studies of therapeutic interventions [51].

### Influence of age, sex, and other factors on NaF uptake

**Fig. 7:**
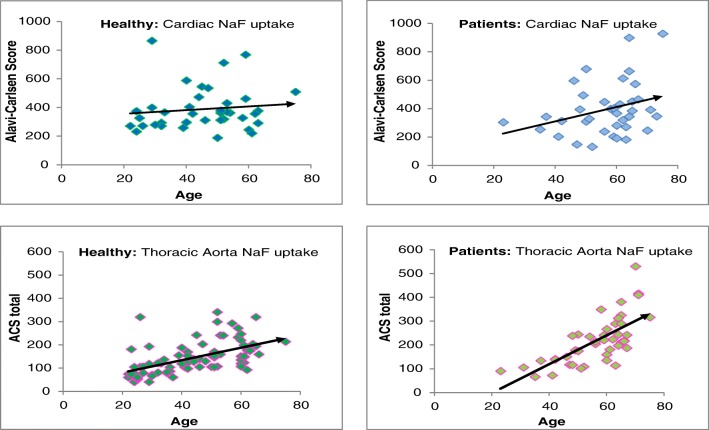
NaF uptake increases with age, the more so in patients, but with a wide scatter. NaF uptake in the heart and thoracic aorta of healthy control subjects and angina pectoris patients as a function of age. Note the steeper slope in angina pectoris patients. All correlations were statistically significant, but with a large scatter indicating that among both healthy individuals and cardiac patients, there are some individuals with very low and some with very high NaF uptake (from the CAMONA material of references 11 and 23)

Most studies examining the association between arterial uptake of NaF and age found a modest, but statistically significant, increase with age which was more pronounced in cardiovascular patients, albeit with a substantial variation over the entire range of adult ages (Fig. 7) (Table 3, Supplementary material). Across methods, there was the mentioned finding that men and women of the same age have roughly the same degree and frequency of abnormal arterial wall NaF uptake [5, 26, 30, 31]. The reason for this is unknown, except for the observations by McGill and co-workers suggesting a difference in time between women and men in the transition from micro- to macrocalcification [47]. Finally, there may be an “osseous exchange” between NaF uptake and bones, meaning that patients with osteoporosis may have a diminished tendency to accumulate NaF in their arteries [25].

### Association between NaF uptake and cardiovascular risk factors

The general finding was a positive correlation between NaF uptake and cardiovascular risk in all examined parts of the arterial system (coronary arteries, the aorta and the carotid, iliac and femoral arteries), a correlation that was significant, but with a wide scatter. No one has investigated the potential superiority or non-inferiority of NaF-PET with regard to risk prediction. A significant association was present in patients with atherosclerosis of varying degrees and patients with oncologic diseases as well as healthy asymptomatic control subjects, albeit with a slightly steeper slope in patients. A similarly significant relationship was not found between FDG uptake and cardiovascular risk; in fact, in some studies, such association could not be proven [23, 31].

### NaF uptake and disease progression

There was a complete lack of longitudinal studies using repeat NaF-PET imaging to investigate the development of NaF-avid arterial wall uptake. A few studies compared initial NaF imaging results with clinical findings at 1–2 years of follow-up, but did not repeat NaF-PET/CT imaging. They reported higher NaF uptake in non-calcified than calcified plaques at baseline and that initial NaF uptake did not correlate with CT-calcification measured in HU or volume a year later [37, 39].

### Methodology

Each center used approximately the same methodology in all their studies. Across centers and institutions, there was great diversity in terms of equipment and acquisition protocol and especially with regard to analysis and quantification methods making direct comparisons virtually impossible. A minority of studies mentioned or listed medication and not a single one examined effects of medication on NaF uptake. There was no consensus on how to measure arterial wall NaF uptake. The majority of centers used some kind of TBR to quantify NaF uptake, i.e., an approach with certain disadvantages [16, 27], other used subtraction of blood background, most often from the superior vena cava or the right atrium, since the recorded NaF concentration is lower here than elsewhere in the venous system, probably depending on cross-talk from NaF uptake in close by bones [28, 30]. A more in-depth discussion of quantification methods is beyond the scope of this article. Instead, readers are referred to articles dealing with such topic [16, 26–29, 52–57].

### Limitations

This review is based on a systematic literature search covering the years 2010–2018, meaning that the 2019 literature is not included and debated. A number of circumstances make the literature on NaF-PET in arteriosclerosis so heterogeneous that it is not possible to draw any firm conclusions. Among these circumstances were differences in image acquisition and acquisition timing, processing, and measures of arterial wall uptake, without and with background correction. Moreover, there was a frequent lack of specific aims for the conducted studies, and shortcomings of the PET technique with its limited spatial resolution were by some considered overcome or not mentioned at all. Nonetheless, a number of observations, listed below, may hold true, whereas other questions remain unanswered. Most 2019 papers on NaF-PET imaging focus on unstable or what is designated vulnerable plaque morphology and detection in predominantly the coronary arteries often employing advanced methodologies [49, 56, 58–63]. In August 2019, the first study on the efficacy of therapy judged by NaF-PET imaging appeared. In a randomized trial of patients with high-risk coronary plaque, there was no effect of dual antiplatelet therapy with ticagrelor in terms of a reduction in plasma tropinin concentration or NaF uptake in the proximal parts of the coronary arteries [50]. The study is a good example of the potential of NaF-PET combined with CT angiography to track the effects of therapeutic intervention on focal NaF uptake to phenotype coronary plaque.

The authors of this review consider measures of focal NaF uptake in the coronary arteries as very promising, but we urge caution, mainly because it is challenging to detect and measure by PET the NaF uptake in anything but the larger, most proximal parts of the coronary arteries [57, 64], which may not be representative for the total cardiac atherosclerotic burden. Thus, we share the opinion of authors Arbab-Zadeh and Fuster that the atherosclerotic burden rather than the “vulnerable plague” should be the target of molecular atherosclerosis imaging [48, 65, 66]. To discuss these important issues in detail is not possible within the frame of this review.

### Information gleaned from the literature

**Fig. 8:**
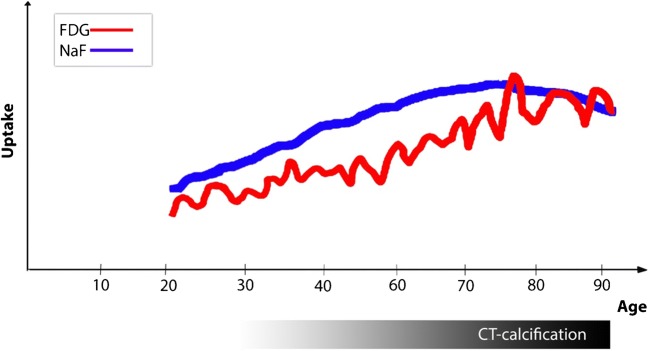
Hypothetical illustration of the possible time- and age-related relationship among arterial wall FDG uptake, NaF uptake, and CT-visible calcification. The courses in childhood and very high ages are unknown. FDG uptake may be a frequently occurring repetitive process throughout life in response to minor or major arterial injuries [44]. By targeting microcalcification [17], NaF appears to be a more persistent marker of early phase atherosclerosis [4, 15, 24, 30, 36] and, through surface adsorption to macrocalcification [17], to a lesser degree also of still ongoing calcification in CT-visible calcifications [24]. The three processes follow different patterns, a slow and protracted increase of FDG uptake, a similarly early in life occurring NaF uptake that tends to persist and increase for some age decades until it decreases when macrocalcification grows and stabilizes, and finally, and in contrast, CT-detectable calcification that appears later in life and continues with aging (illustration by Dr. Reza Piri, Dept. of Nuclear Medicine, Odense University Hospital, Odense, Denmark)

Despite reservations and shortcomings, the following information could be gleaned from the existing literature. In addition, we have made a hypothetical figure illustrating the potential time course of FDG-, NaF-, and CT-detectable arterial wall changes (Fig. 8).

- NaF is a highly specific and sensitive PET tracer targeting microcalcification, often due to tissue necrosis but not being a component of natural arterial wall tissue.
- NaF uptake indicates and represents active, ongoing microcalcification.
- NaF uptake is often present and higher in areas without than with CT-calcification and coincides only sporadically with arterial foci of abnormal FDG uptake.
- NaF uptake associated with CT-calcification is surface located and less when the density of CT-calcification is high.
- NaF uptake is higher in vulnerable, high-risk, and ruptured plaques, but cannot identify these or predict events in individual patients with a high probability.
- NaF uptake increases slightly with age, but with a large variability, indicating that some subjects are more inclined to develop atherosclerosis than others.
- NaF uptake does not exhibit the same 10-year delay in women compared with men as observed with regard to CT-visible macrocalcification and cardiovascular events.

Answers to the following questions have not been provided thus far:

- Do fatty streaks in human arteries accumulate NaF? If so, what component of streaks does so?
- Does NaF- (and/or FDG-) avid microcalcification progress to CT-visible macrocalcification in the natural progression of atherosclerosis?
- In which compartment of the arterial wall does microcalcification first appear?
- Can NaF-avid microcalcification be diminished or abolished by medical therapy or other types of intervention? This is especially important because statin therapy promotes macrocalcification [67].
- If so, should therapy be initiated before a certain critical time point to be effective?
- Is atherosclerotic disease burden in the arterial system a better predictor of cardiovascular events than uptake in selected high-risk or vulnerable plaques?
- Is atherosclerotic burden by NaF-PET in individual patients equal or superior to common risk factors with regard to prediction of future cardiovascular events?

## Conclusion

The available literature on NaF-PET imaging of atherosclerosis is limited, heterogeneous, and diverging with regard to scope, size, methodology, and studied arterial segment, and devoid of long-term follow-up studies and intervention trials. Early atherosclerosis is characterized by arterial wall microcalcification that is detectable and quantifiable by means of NaF-PET/CT probably years or decades before it presumably gives rise to CT-visible macrocalcification, which may take up NaF on its surface, but with decreasing intensity the more dense the calcification. NaF is a highly specific and very sensitive tracer with high affinity for active, ongoing molecular microcalcification that appears to be due to tissue necrosis. NaF-PET is the only existing in vivo modality that can detect and quantify the active atherosclerotic process. NaF uptake correlates consistently with cardiovascular risk factors, suggesting that NaF-PET imaging alone or in conjunction with selected risk factors is capable of providing a better, much earlier, and more accurate assessment of the arteriosclerotic burden in the body than what can be achieved with other techniques. Future research should document translation of NaF-avid microcalcification into CT-visible macrocalcification and assess to what extent and when anti-atherosclerotic intervention can diminish or abort the arteriosclerotic disease process.

## Electronic supplementary material

ESM 1 (DOCX 55 kb)
