# Supplementary material for: Atherosclerosis imaging with 18F-sodium fluoride PET: state-of-the-art review
Source: Eur J Nucl Med Mol Imaging. 2019 Nov 27;47(6):1538–51. doi: 10.1007/s00259-019-04603-1 (PMC7188711; doi:10.1007/s00259-019-04603-1)
Supplement: Supplementary file 1 — (DOCX 55 kb) [file 259_2019_4603_MOESM1_ESM.docx]

**Supplementary Material**

Contents

**Table 1 Studies on early detection and/or prevalence2**

**Table 2 Studies on NaF uptake in vulnerable, high-risk or ruptured plaque 4**

**Table 3 Studies on influence of age, sex and other factors 6**

**Table 4 Studies on arterial NaF uptake and cardiovascular risk factors8**

**TABLE 1 Studies on early detection and/or prevalence**

| **First author**  **(Ref. #)** | **Patients, n (female)**  **Age** | **Material**  **pro- or retrospec­tive** | **Tracer** | **Arterial segment** | **Purpose** | **Main findings** | **Comment** |
| --- | --- | --- | --- | --- | --- | --- | --- |
| Derlin et al. 2010 (4) | 75 (48)  65.2±12.3 y | Pts referred for exclusion of bone metastases.  Retrospective. | NaF | Carotids  thoracic & abdominal aorta, Iliac, femoral. | Prevalence, distribution, topographic relationship of NaF uptake and CT calcification. | NaF uptake at 254 sites in 57 (76%) pts. Calcification at 1,930 sites in 63 (84%) pts. Co-localization of NaF accumulation and calcification in 223 areas of uptake (88%). Only 12% of all arterial calcification sites showed NaF uptake in at least some part. Prevalence of CT calcification highest in abdominal aorta, femoral and iliac arteries. | First NaF-PET in atherosclerosis in humans. Insufficient material description. Convincing feasibility study.  SQS = 18 |
| Derlin et al. 2011 (44) | 45 (24)  57.7±13.7 y, range 21.6–80.6 | Oncologic pts.  FDG and NaF studies performed 1.2 months apart (range 0.1-4.8).  Retrospective. | NaF & FDG | Carotids, aorta (4 parts), femoral arteries. | Compare inflammation (FDG uptake) and ongoing mineral deposition (NaF uptake) with calcified plaque burden assessed by CT. | NaF uptake at 105 sites in 27 (60%) of the 45 study pts. Mean TBR was 2.3 +/- 0.7. FDG uptake at 124 sites in 34 (75.6%) pts, and mean TBR 1.5 +/- 0.3. Calcified atherosclerotic lesions at 503 sites in 34 (75.6%) pts. Eighty-one (77.1%) of the 105 lesions with marked NaF uptake and only 18 (14.5%) of the 124 lesions with FDG accumulation were co-localized with arterial calcification. Coincident uptake of both NaF and FDG in only 14 (6.5%) of the 215 arterial lesions with radiotracer accumulation. | First study describing differences in location of FDG uptake, NaF uptake and CT-calcification.  SQS = 19 |
| Beheshti et al. 2011 (26) | 51 (34)  29-90 y | Oncologic pts.  Retrospective. | NaF | Heart and 2 3 cm long segments of thoracic and abdominal aorta. | Prevalence of NaF uptake in heart and aortic wall and association with age. | Significant positive linear correlation between uptake and age in the heart and aorta (with a wide scatter), demonstrated by uptake in five age groups (≤40, 41-50, 51-60, 61-70 >70 y). Creatinine was no confounder. No or minimal CT-calcification noted in heart, some in the aorta, no NaF foci were directly visible. The ≤40 age group had Naf uptake in the heart of 57% of that of the >70 age group and in the aorta of 65% of that of the >70 age group: is there an adult age without NaF uptake? | Use of cardiac global molecular calcification score, GMCS and of SUVmean in the aorta.  SQS = 17 |
| Li Y et al. 2012 (33) | 61 (3)  58 (27-91) y | Pts imaged for bone lesions from cancer and other diseases.  Retrospective. | NaF | Major coronaries, thoracic and abdominal aorta, femoral. | NaF uptake vs CV history and risk factors. | NaF uptake in 361 vascular territories of 59 pts (97%), calcifications in 317 of 49 (88%) pts. NaF uptake more frequent in aorta and femoral arteries, calcification more frequent in carotids and coronaries than NaF uptake. No consistent NaF–calcification overlap, doubtful correlation btw proximal coronary NaF uptake and CV risk, but correlation with history of CV events. | Mixture of blood background. SUVmax for 1 cm circular ROIs in the target artery wall. Assessment of CV risk not specified.  SQS = 12 |
| Janssen et al. 2013 (35) | 409 (233) 63.5 (19.7-90.8) Y | Oncologic pts.  Retrospective. | NaF | Femoral (linear calcifications) & carotids, aorta, iliac and femoral. | Can NaF visualize and quantify diffuse media mineral deposition? Does this type of tracer uptake correlate with risk factors and plaque burden representing intimal disease in other vascular territories? | Linear NaF uptake in femoral arteries of 159 (38.9%) pts, 73 (45.9%) of these with medial-type linear CT calcifications. CT-visible arterial plaques in carotids, aorta, right and left iliac, right and left femoral arteries present at 3,767 sites in 340 (83.1%) of the 409 pts, highest in the abdominal aorta, followed by iliac arteries and thoracic aorta. NaF uptake in femoral arteries correlated significantly with age, hypertension, hypercholesterolemia, and diabetes, history of smoking and prior CV events. Prevalence of linear femoral NaF uptake increased with number of CV risk factors from 9.7% of cases in group with 0 risk factors to 64.1% in group with ≥ 5 risk factors | SQS = 21 |
| Quirce et al. 2013 (18) | 15 (5)  52-82 y | Pts with carotid plaque by contrast CT; 8 + & 7 - neurovascular symptoms.  Prospective. | NaF | Carotids. | Assessment of NaF uptake in carotid plaques. | A total of 29 plaques, 19 asymptomatic and 10 symptomatic ones. All had NaF uptake, with level ≥2 in all symptomatic plaques, but <2 in 6 of the asymptomatic ones. | Visual analysis only. Only slight association with neurological symptoms.  SQS = 11 |
| Fiz et al. 2015 (24) | 64 (50) 65.3±8.2 y (26-81) | Pts with breast or prostate cancer. All pts with ≥1 CT visible calcification (= density ≥ 130 HU).  Retrospective. | NaF | Infrarenal abdominal aorta. | Assessment of whether early stage CT visible calcification has higher NaF uptake and is thus more actively growing than end-stage calcification. | All 64 had ≥1 light plague (130-210 HU), 41 (64%) had medium plaque (211-510 HU) and 31 (48%) heavy plaque. Non-calcified arterial hot spots were present in 55 (86%) pts. Light plaque had highest NaF TBR of 2.21 vs medium 1.59 vs heavy 1.14. Heavy plaque uptake was as low as uptake in control regions (1.16). Uptake in non-calcified hot spots was the highest: 3.89 vs 2.21 in light plaques, i.e. 76% higher. Inverse relation btw plaque density and TBR. | TBR was compared to TBR of a control region (arterial segment without CT calcification or increased NaF uptake.  SQS = 21 |
| Quirce et al. 2016 (19) | 9 (1)  50-83 y | Pts with recent cerebrovascular attack; 9 symptomatic and 9 asymptomatic plaques.  Prospective. | NaF & FDG | Carotids. | Not clearly specified. | All plaques had NaF and FDG uptake. Higher NaF uptake by 11/18 plaques, 6 symptomatic and 5 asymptomatic. Mean NaF TBR was 2.12 +/- 0.44 in symptomatic vs 1.85 +/- 0.46, p=0.22 in asymptomatic group. Overall, higher NaF than FDG uptake in symptomatic plaque; NaF uptake highest for low and lowest for high calcium content. Active calcification and inflammation are simultaneous processes in symptomatic and asymptomatic carotid atheroma. Active calcification seems predominant over inflammation in both groups. | Visual assessment plus various TBRs.  SQS = 13 |
| Kitagawa et al. 2017 (13) | 32 (6)  66±8 Y | Pts with known or suspected CAD and ≥1 CT- visible coronary lesion.  Prospective. | NaF | Coronary arteries. | Relation of NaF uptake to CT visible coronary lesions. | Calcified plaque (CP) (≥130 HU), non-calcified plaque (NCP) and partially calcified plaque (PCP). High risk NCP and PCP defined by high remodeling index (1.1) and low density (<30 HU). Per patient, logarithmically transformed coronary calcium score correlated with TBRmax; 15 pts with MI or unstable AP had higher TBRmax than those without: 1.36 vs 1.15, p=0.0006). Per lesion, PCP had higher TBRmax than CP and NCP; 1.17 vs 1.00 and 0.92, p<0.0001; lesions with high risk features had higher TBRmax; 1.20 vs. 1.02, p=0.001. | Not very clear study.  SQS =15 |
| Ferreira et al. 2018 (31) | 25 (11) 63.9±8.6 y | Pts with 10-y risk of fatal CV event ≥5%. No known CVD, some with “very abnormal single-risk factor”.  Prospective. | NaF | Coronaries, carotids, aorta, but not clearly defined. | NaF uptake in relation to gender and number of CV risk factors. No hypotheses. | 131 lesions in total, 71 of which also calcified. 96% of pts had at least one lesion with NaF uptake. Diverging results with TBR vs. blood-background corrected uptake per lesion (CUL). Of note: 1) NaF uptake was similar in the two sexes despite same age level (approx. 64 y in both); this goes for number of plaques in three territories and the mean TBR significantly, but only marginally higher in men than women (1.8 vs. 1.7), whereas the blood-background corrected CUL was not: 0.7 vs. 0.6. | Strange subtraction of blood SUVmean from SUVmax values. New is focus on high CV risk subjects.  SQS =15 |

CAD = coronary artery disease; CV = cardiovascular; FDG = 18F-fluorodeoxyglucose; HU = Hounsfield unit; NaF = 18F-sodium fluoride; pts = patients; SQS = subjective quality score; SUV = standardized uptake value; TBR = target-to-background ratio.

**TABLE 2 Studies on NaF uptake in vulnerable, high-risk or ruptured plaque**

| **First author**  **(Ref. #)** | **Patients, n**  **(female)**  **Age** | **Material** | **Tracer** | **Arterial segment** | **Purpose** | **Main Findings** | **Comments** |
| --- | --- | --- | --- | --- | --- | --- | --- |
| Joshi et al. 2014 (34) | 40 (37)  62±8  40 (36)  67$\pm8$ | Pts with acute MI  Pts with stable angina | NaF & FDG | Coronary arteries and carotid artery specimens | Investigate if NaF uptake compared with FDG uptake can identify ruptured and high-risk atherosclerotic plaques in pts with symptomatic coronary and carotid artery disease. | In 37/40 (93%) MI pts slightly, but significantly, higher median NaF TBRmax was seen in culprit vs non-culprit lesions (defined how?): 1.66 vs 1.24. Coronary FDG uptake was often obscured and when discernible there was no difference between uptake in culprit and non-culprit lesions (1.71 vs 1.58). NaF uptake noted at sites of all carotid plaque ruptures and plaques with histological evidence of active calcification (?), macrophage infiltration, apoptosis and necrosis. 18 (45%) SAP pts had plaques with focal NaF uptake that were associated with more high risk features on intravascular ultrasound than those without uptake. | Coronary FDG uptake was often obscured by myocardial uptake and where discernable there was no significant difference between uptake in culprit vs. non-culprit coronary lesions.  SQS = 15 |
| Adamson et al. 2015 (10) | 40 with acute MI + 40 with stable angina. Age and gender not specified. | In addition, 9 carotid specimens from carotid endarterectomy. Prospective, but Imaging for other purposes. | NaF & FDG | Coronaries | Not stated. | 1. Positive plaque if TBRmax ≥25% than reference non-culprit lesion of MI pts. Was so with NaF in 37/40 (93%).  2. Virtual histology IVUS showing high risk feature in 18/40 pts with abnormal NaF Uptake.  3. Increased NaF uptake in regions of macroscopic plague rupture.  4. FDG analysis compromised by myocardial uptake obscuring the coronary arteries. | Commentary probably based on the above paper. Speculates with no subjective standards, unclear methods, no solid data and no documentation that NaF characterizes vulnerable plagues.  SQS = 7 |
| Lee et al. 2017 (12) | 51 (6)  62.3±8.2 y | Pts with NaF PET before invasive coronary angiography, Prospective. | NaF | Coronaries | To evaluate “the clinical relevance” of NaF uptake using optical coherence tomography (OCT), IVUS and CT-angiography. | NaF TBRmax was higher in pts with high-risk than non-high-risk plaque judged by IVUS (1.09 vs 0.62, p=0.001) and in pts with high -risk than non-high-risk judged by OCT (0.76 vs 0.42, p=0.014). A total of 14/15 of plaques with high-risk by both IVUS and OCT had positive NaF uptake. | Proximal coronary arteries only.  SQS = 15 |
| Vesey et al. 2017 (21) | 26 (10)  71.7±12.3 (66.1±12.5) | After recent transient ischemic attack or minor stroke: 18 with culprit carotid stenosis awaiting  endarterectomy and 8 controls without culprit carotid atheroma.  Prospective. | NaF & FDG | Carotids | Assessment of whether NaF or FDG can identify culprit or high-risk carotid plaque. | On histological and micro PET/CT analysis, NaF selectively highlighted microcalcification. Carotid NaF uptake was increased in clinically adjudicated culprit plaques vs asymptomatic contralateral plaques (log_10_SUVmean 0.29±0.10 versus 0.23±0.11, P=0.001) and compared with control patients (0.29±0.10 versus 0.12±0.11, P=0.001). NaF uptake correlated with high-risk plaque features and “predicted” cardiovascular risk [r=0.65, P=0.002]). Carotid FDG uptake appeared to be increased in 7 of 16 culprit plaques, but no overall differences in uptake were observed in culprit vs contralateral plaques or control patients. FDG correlated with predicted cardiovascular risk (r=0.53, P=0.019), but not with plaque phenotype. | Higher uptake of NaF alone does not allow for identification og culprit or high-risk plaque. This requires demonstration (as not done) that NaF-PET/CT can with ≥85% probability identify them. Logarithmic SUVs minus 1.96 SD yield close to zero or negative numbers.  SQS = 19 |
| Kitagawa et al. 2018 (14) | 32 (6)  66±9 y | Coronary lesions analyzed with cardiac CT and NaF-PET/CT. During 2-y follow-up: 1 MI, 3 UA and 7 late coronary events.  Prospective. | NaF | Coronaries | NaF PET/CT for prediction of coronary events and NaF + CT for combined use in coronary risk assessment. | Significant, but small, differences among pts with and without later “coronary events”. Pts with an M-TBRmax ≥1.28 had a higher risk of “earlier coronary events”, making the authors conclude that NaF PET/CT has the potential to detect high-risk coronary disease and individual coronary lesions and predict future coronary events when combined with cardiac CT. | Late coronary revascularization is a questionable endpoint. Dubious statement (page 2) in the article “that data, analytic methods, and study material will not be made available to other researchers…etc.”  SQS = 14 |
| Li L et al. 2018 (15) | 32 (10): 30 unstable aged 57±8 plus 2 stable aged 65±2 y | Symptomatic CAD pts studied with NaF-PET and IVUS within 2 days.  Prospective. | NaF | Coronaries | Coronary NaF uptake compared with IVUS in pts with symptomatic CAD. | Higher NaF uptake in fibrocalcific (1.42) than thincap with spotty calcifications (1.32) and thickcap mixed atheroma (1.28), while normal (0.96) in fibrotic plaques. NaF uptake most likely localized in the border zone of intensive calcification. NaF uptake associated with increase high-risk plaque features on IVUS. | SQS = 19 |
| Marschesseau et al. 2018 (16) | 10 (1)  48±7 y | Pts with STEMI examined after 9-24 d with PET/MRI and PET/CT  Prospective. | NaF | Coronaries | Detection of high risk coronary atheroma by NaF-PET/CT and quantification of myocardial scar tissue to “add understanding of the common processes between infarction and atheroma.” | Joshi method of coronary ROI determination of TBRmax and TBRmean for myocardial (scar) uptake.^47^ TBRmax was significantly higher in culprit than non—culprit lesions 2.11 vs 1.36 and in scar compared to remote myocardial tissue, 0.87 vs 0.72. | Good remarks about limitations of TBRmax.  SQS = 14 |
| Hop et al. 2018 (20) | No pts. Experimental study of carotid tissue samples from 23 stroke pts. | 17 culprit and 6 non-culprit lesions from 23 pts and 15 renal arteries samples from healthy kidney donors.  Prospective. | NaF | Carotids | 1) MicroPET NaF in culprit and non-culprit carotid plaques vs samples of non-calcified renal arteries.  2) MicroNaF uptake vs. micro CT uptake. | Average NaF uptake expressed as a SUVmean value of chosen ROI was equally high in culprit and non-culprit lesions, ie. 2.32 %Inc/g vs 2.35, but 5 times as high as in normal renal arteries (2.32 vs. 0.44). Only a median of 10% of CT calcification VOI showed Increased NaF uptake and only merely a median of 35% of NaF PET VOIs showed calcification on CT. | Important and nice experimental study showing similar NaF uptake in specimens of culprit and non-culprit lesions of carotid arteries from high-risk patients.  SQC = 23 |

CAD = coronary artery disease; CV = cardiovascular; FDG = 18F-fluorodeoxyglucose; HU = Hounsfield unit; IVUS = intravascular ultrasound; MI = myocardial infarction; NaF = 18F-sodium fluoride; OCT = optical coherence tomography; pts, patients; STEMI = ST elevation myocardial infarction; SQS = subjective quality score; SUV = standardized uptake value; TBR = target-to-background ratio.

**TABLE 3 Studies on Influence of age, sex and other factors**

| **First author**  **(Ref. #)** | **Patients, n**  **(female)**  **Age** | **Material** | **Tracer** | **Arterial segment** | **Purpose** | **Main findings** | **Comments** |
| --- | --- | --- | --- | --- | --- | --- | --- |
| Derlin et al. 2011 (5) | 269 (166)  66.1±12.4 y | Neurologically asymptomatic oncologic pts.  Retrospective | NaF | Common carotids | Correlation of NaF uptake with CV risk factors and calcified plaque burden | NaF uptake at 141 sites in 94 (34.9%), always co-localized with CT-calcification, thus no PET+ and CT- carotid sites! NaF uptake associated with age, male sex, hypertension, hypercholesterolemia, but not with history of smoking, diabetes, obesity or prior CV events. Calcified plaques not associated with age, male sex or obesity. NaF SUVmax was associated with number of risk factors, TBR not! | Carotid NaF uptake more frequent in pts with high risk profile; weaker correlated with risk factors than calcified plaque burden.  SQS = 18 |
| Beheshti et al. 2011 (26) | 51 (34)  29-90 y | Oncologic pts.  Retrospective | NaF | Heart and two 3 cm long segments of thoracic and abdominal aorta | Prevalence of NaF uptake in heart and aortic wall and association with age | Naf uptake in whole heart and aorta increased significantly with mean age in five age groups (≤40, 41-50, 51-60, 61-70. >70), albeit with a wide scatter. Creatinine was no confounder. Minimal or no CT-calcification noted in heart, some in the aorta, NaF uptake was not directly visible. The ≤40 age group had a whole-heart NaF uptake that was 57% of that of the >70 age group and an aortic uptake 65% of the >70 age group: is there an adult age without NaF uptake? | First use of cardiac global molecular calcification score, GMCS. Use of SUVmean in aorta.  SQS = 17 |
| Blomberg et al. 2014  (27) | 20 (10) HCs  41.8±14.5 y and  18 (9) APs  54.7±13.0 Y | Healthy controls (HCs) and angina pectoris pts (Aps)  Prospective | NaF | Heart and thoracic aorta | Does maximizing contrast btw arterial wall and blood pool improve arterial wall calcification metabolism? | 45, 90, 180 min acquisition time points. cSUVmax & TBR.  Both 45 and 90 acquisition time pointe are acceptable. | Inter- and interrater agreement in 10 subjects.  TBR calculation inferior to cSUVmax!  SQS = 22 |
| Derlin et al. 2015 (25) | 304 (172) 62.4 y (19.7-90.8) | Pts referred for exclusion of bone metastases.  Retrospective. | NaF | Femoral | NaF uptake compared with CV risk factors, calcified plaque burden, and regional bone metabolism as assessed by PET/CT. | 608 femoral arterial segments in 304 pts. Inverse correlation btw arterial NaF and regional bone metabolism. TBR for femoral arteries increased with age, and as total calcified plaque burden (CPB) increased; CV risk factors increased and correlated with male gender, hypertension, hypercholesterolemia, diabetes and prior CV events.  Overall conclusion was that arterial mineral deposition increases with age while regional bone metabolism decreases – first time this was done in the same pts. | Very relevant – first NaF artery wall uptake vs bone calcification content and age in the same (oncologic) pts.  SQS = 20 |
| Blomberget al. 2015 (28) | 89 (42)  44±14 y  21-75 y | Healthy control subjects.  Prospective. | NaF | Heart and thoracic aorta | How does blood activity, renal function, injected dose, circulation time affect quantification of arterial NaF uptake? | Arterial NaF uptake (SUVmax) is affected by blood activity, injected dose and PET/CT system.  Is it better to give a standard dose of NaF instead of a dose per kg body weight? | Blood NaF activity lowest in superior v. cava.  NaF SUVmax values in healthy controls.  SQS = 22 |
| Blomberg et al. 2017 (30) | 89 (42)  21-75 y | Health control subjects.  Prospective. | NaF & FDG | Thoracic aorta, carotids and co­ro­naries. | Determine age- and sex-specific reference values and influence of age and sex on FDG and NaF uptake | FDG and NaF uptake (measured as blood pool subtracted SUVmax) increased with age. With FDG this was significant in the descending part of the thoracic aorta only (+37% from age 20-29 to age ≥60 in women and +19% in men); with NaF it was significant in the ascending aorta (+21% in women and +26% in men), aortic arch (+19% in women and +37% in men), descending thoracic aorta (+25% in women and +47% in men), and coronary arteries ((+8% in women and +30% in men). NaF uptake was not influenced by sex, but increased more with age and was somewhat higher in men. | First data on arterial FDG and NaF uptake in healthy individuals. Surprisingly high uptake values in young individuals: methodology problems? Partial volume correction is called for.  SQS = 19 |
| Ferreira et al. 2018 (31) | 25 (11) 63.9±8.6 y | Pts with no known CV disease, but a calculated ≥5% 10-year risk of a CV event. | NaF | Coronaries, carotids, aorta, but not clearly defined. | NaF uptake in relation to gender and number of CV risk factors. No hypotheses. | 131 lesions in total, 71 of which also calcified. 96% of pts had at least one lesion with NaF uptake. Diverging results with TBR vs. blood-background corrected uptake per lesion (CUL). NaF uptake was similar in the two sexes despite same age level (approx. 64 y in both); this goes for number of plaques in three territories and the mean TBR significantly, but only marginally higher in men than women (1.8 vs. 1.7), whereas the blood-background corrected CUL was not: 0.7 vs. 0.6. | Strange subtraction of blood SUVmean from SUVmax values. New is focus on high CV risk subjects. Lack of sex difference also seen by Blomberg et al.^27^ SQS = 15 |

CAD = coronary artery disease; CV = cardiovascular; FDG = 18F-fluorodeoxyglucose; HU = Hounsfield unit; NaF = 18F-sodium fluoride; pts, patients; SQS = subjective quality score; SUV = standardized uptake value; TBR = target-to-background ratio.

**TABLE 4 Studies on arterial NaF uptake and cardiovascular risk factors**

| **First author**  **(Ref. #)** | **Patients, n**  **Age, Mean±SD or range** | **Material** | **Tracer** | **Arterial segment** | **Purpose** | **Main findings** | **Comments** |
| --- | --- | --- | --- | --- | --- | --- | --- |
| Dweck et al. 2012 (9) | 119 (38)  72±8 y, | Volunteers with (66%) and without aortic stenosis. 66% with aortic stenosis. 13 with no history of CAD and no CT calcifications served as controls. Of the remaining 106, 41 had prior CAD and 65 had calcium scores >0. | NaF & FDG | Coronary arteries. | Investigation of coronary arterial NaF and FDG uptake as markers of active plaque calcification and inflammation, respectively. | CT and NaF-PET/CT performed successively on the same day, FDG-PET/CT on a later day. NaF acquisition about 60 in after, FDG acquisition about 90 after tracer injection with a median interval between the two PET scans of 7 days (range 1-14). Correlation between NaF TBR and CAC but not between FDG TBR and CAC score; FDG score was not associated with increased rates of CAD, angina, prior revascularization or previous MACE. CAC score was not correlated with 10-y FRS. NaF uptake in the coronaries not correlated with that in the aorta, which made authors the authors state that blood borne biomarkers may not reflect coronary calcification, but rather large vessel and bone calcification. No data plots showing scatter. | First study on coronary NaF uptake. NaF TBRmax values were low, but highest in the 13 controls (1.61). FDG uptake not possible to quantify in 49% of vessel territories n these pts, who received a carbohydrate poor diet. FDG not very reproducible.  SQS = 13 |
| Janssen et al. 2013 (35) | 409 (233) 63.5 (19,7-90,8) Y | Oncologic patients.  Retrospective | NaF | Femoral; linear calcifications. Carotids, aorta, right and left iliac, right and left femoral arteries | Can NaF visualize and quantify diffuse media mineral deposition? Does this type of tracer uptake correlate with risk factors and plaque burden representing intimal disease in other vascular territories? | Linear NaF uptake in femoral arteries of 159 (38.9%) pts, 73 (45.9%) of these with medial-type linear CT calcifications. CT-plaques in carotids, aorta, right and left iliac, right and left femoral arteries present at 3,767 sites in 340 (83.1%) of 409 pts, highest in the abdominal aorta, followed by iliac arteries and thoracic aorta. NaF uptake in femoral arteries correlated significantly with age, hypertension, hypercholesterolemia, diabetes, history of smoking and prior CV events. Prevalence of linear femoral NaF uptake increased with number of CV risk factors from 9.7% of cases in group with 0 risk factors to 64.1% in group with ≥ 5 risk factors | Largest NaF material to this date.  SQS = 21 |
| Morbelli et al. 2014 (37) | 80 (60)  65.3 y, range 26-81. | Oncologic pts with breast or prostate cancer.  Retrospective. | NaF | Carotid, subclavian, iliac and whole aorta. | Regional association of macrocalcification and NaF uptake | No correlation between regional calcification load and regional TBRmax in any of the studied arterial segments. Visible calcium deposits were dependent on age, but not CV risk factors. NaF correlated with all CV risk factors except BMI. Vascular NaF displays a different regional distribution than macroscopic arterial calcification. | SQS = 14 |
| Fiz et al. 2016 (29) | 77 pts (44) 63 y, range 44-83. | Pts with breast or prostate cancer examined for bone metastases. No history of MACE and no statins.  Retrospective. | NaF | Heart and thoracic aorta (3 segments). | Relationship btw NaF uptake and CV risk | Material split into three risk groups according to FRS; high = FRS >20%, intermedidate 10-20%, low <10%. 20 pts high, 36 intermediate, 22 low, low groups was younger 58.3 y vs medium 65 y vs high 65 y. Clear association between NaF uptake and CV risk in particular between descending aorta or myocardium on one side and CV risk on the other. Correlation between in particular descending aorta NaF uptake and myocardial uptake in line with reference 21 in this article, i.e., Brodov study from Daniel Berman’s group suggesting aortic calcification as a predictor of coronary calcification. | Well conducted and thoughtful study. Use of both TBR and Beheshti et al.^40^  methodology with a global molecular calcification score including both macro- and microcalcification signals as a potentially better harbinger of future events. SQS = 22 |
| Blomberg et al. 2017 (23) | 139 (67), 21-75 y | 89 healthy controls plus 50 angina pectoris pts.  Prospective. | NaF & FDG | Thoracic aorta | Relationship between CVD risk and FDG, NaF and CT. | FRS 3.7 times higher in subject with NaF uptake in the highest quartile compared to those in the lowest quartile and similar to finding in subjects with a thoracic aorta calcium burden on CT in the highest quartile compared to those in the lowest quartile. FRS risk was similar in subjects in all quartiles of thoracic aorta FDG uptake. | Important study putting FDG uptake on the sideline.  SQS = 23 |
| Blomberg et al. 2017 (11) | 89 (42), 21-75 y | 89 healthy controls.  Prospective. | NaF | Heart = coronaries | Relationship between coronary NaF uptake & FRS. | Female sex, age, and BMI were independent factors of increased coronary NaF uptake. Coronary NaF uptake increased linearly with the number of CV risk factors even in healthy controls. | SUVmax and not SUVmean.  SQS = 22 |
| Oliveira-Santoz et al. 2017 (32) | 25 (11)  63.9 ± 8.6 y | High risk hypertensive pts with SCORE > 5% for 10 year risk.  Prospective. | NaF | Carotid and  coronary arteries, thoracic and abdominal aorta. | The proportion of coronary, carotid and aortic plaques with NaF uptake in pts with no clinically apparent CVD, but high CV risk. | Ninety six percent of subjects showed NaF uptake in the aorta (CUL 0.9±0.3), 40% in the carotid arteries (median CUL 0.0, IQR 0.0-0.7) and 64% in the coronary arteries (0.4, IQR 0.0-0.6). Individuals with ≥ five risk factors (60%) had increased overall NaF uptake (1.1±0.3 vs. 0.7±0.3, p < 0.01), which was positively correlated with predicted fatal CV risk SCORE (r = 0.49, p = 0.01). No correlation between coronary NaF uptake and calcium score (p = 0.87). Thoracic fat was moderately correlated with overall CUL (r = 0.41, p = 0.04). | Heavy medication. Atherosclerotic plaque NaF uptake was related to CV risk factors and thoracic fat volume, but no association between coronary NaF uptake and calcium score.  SQS = 14 |

CAC = coronary artery calcium; CAD = coronary artery disease; CUL = (superior vena cava blood activity) corrected uptake per lesion; CV = cardiovascular; FDG = 18F-fluorodeoxyglucose; FRS = Framingham Risk Score; HU = Hounsfield unit; MACE = major adverse cardiovascular events; IQR = interquartile range; NaF = 18F-sodium fluoride; pts, patients; SCORE = Systematic COronary Risk Evaluation (European Heart Association); SQS = subjective quality score; SUV = standardized uptake value; TBR = target-to-background ratio.
